# Are we speaking the same language? Call for action to improve theory application and reporting in behaviour change research

**DOI:** 10.1186/s12889-021-10541-1

**Published:** 2021-03-10

**Authors:** Taylor Willmott, Sharyn Rundle-Thiele

**Affiliations:** Social Marketing @ Griffith, Griffith University, 170 Kessels Road, Nathan, QLD 4111 Australia

**Keywords:** Behaviour, Health, Intervention, Framework, Public health, Standardise, Theory

## Abstract

Inconsistencies are evident in definitions and interpretations of theory, application of theory, and reporting of theory use within the behaviour change field impeding cumulative knowledge advancement. Standardised frameworks and methods are needed to support the definition, application, and reporting of theory, and to assist researchers in understanding how theory should be applied to build cumulative knowledge over time. Progress is being made with the development of ontologies, taxonomies, methods for mapping interventions, and coding schemes; however, consolidation is needed to improve levels and quality of theory use, and to facilitate the translation of theory-driven research in practice. This paper discusses the importance of rigorous theory application and reporting in health-related behaviour change research and outlines the need for a standardised framework that supports both researchers and practitioners in designing, implementing, and evaluating theory-driven interventions in a concrete and consistent manner. To this end, several recommendations are provided to facilitate the development of a standardised framework that supports theory application and reporting in the behaviour change field. Concrete and consistent theory application and reporting will permit critical appraisal within and across studies, thereby advancing cumulative knowledge of behaviour change over time.

## Main text

Human behaviour lies at the heart of society’s most pressing issues. A salient example is the Coronavirus Disease 2019 (COVID-19) pandemic. In the absence of widespread dissemination and uptake of efficacious vaccines, reducing the transmission of COVID-19 and halting the spread of the virus requires rapid, extensive, and lasting behaviour change to enact protective behaviours (e.g. hand hygiene, covering coughs and sneezes, wearing face masks, staying home if you feel sick, and adhering to social distancing measures across communities) [1]. Noncommunicable disease, often referred to as chronic disease, is another pressing public health crisis occurring globally [2]. Rising rates in overweight and obesity, metabolic syndrome, and associated chronic disease, have been attributed to an increase in energy dense nutrient poor diets, decreased levels of physical activity, and increased levels of sedentary behaviour [3–5]. Even small changes in high-risk behaviours such as physical inactivity, sedentary behaviour, and poor diet could have a substantial impact on individual and population health outcomes [2]. Consequently, understanding how to effectively change behaviours at both the individual and population level is important and urgent [6, 7]. By linking activities with outcomes, behavioural interventions can provide a more nuanced understanding of how the desired outcomes in individual and/or population health are being achieved (or not being achieved) [8].

Despite promising findings for the effectiveness of health-related behaviour change interventions, the systematic accumulation of evidence and guidance regarding how to develop effective, efficient, and scalable interventions remains slow [9]. Intervention findings reported are often highly variable, with many failing to achieve the desired outcomes [9]. Moreover, many are unable to be delivered at the scale required to bring about population-level changes in health outcomes [9]. Understanding health behaviours, along with the characteristics of interventions intended to influence them, is fundamental in building a road map that can be reliably drawn upon to elicit the desired outcomes in individual and/or population health, thereby reducing avoidable morbidity and mortality [10, 11]. To facilitate the development of a cumulative knowledge base that can be reliably accessed to understand how to deliver beneficial change across different behaviours, populations, and contexts, researchers and practitioners must adopt a *theory-driven* approach that leverages the known benefits of theory use across the *complete* intervention life cycle.

### Definitions and interpretations of theory

The term “theory” has been defined in many varied ways [12], often to the detriment of the field’s cumulative knowledge base. Within both the behavioural and social sciences several terms (e.g., orientation, concept, framework, model, logic model, theory of change, system of organisation, and so forth) are often used interchangeably with theory, and there remains a lack of generally accepted terms to appropriately guide terminology use among researchers and practitioners to support the consolidation of evidence and advancement of cumulative knowledge [10]. Variable term use may, in part, reflect differing philosophical orientations. Depending on philosophical stance, scholars will hold different views regarding what constitutes theory. For some, theory represents a set of assertions or propositions, an abstract conceptualisation of the relationships between entities, or as a general principle that is applied to explain or predict phenomena [13]. For others, theory refers to the verification of facts, systems of organisation, law-like generalisations, and tested hypotheses [13]. Broadly speaking, scientific theory may be viewed as an organised way of thinking about observed phenomena; and when applied, can reveal deeper understandings of how and why things occur the way they do [13].

The lack of clarity surrounding what constitutes theory in the behavioural and social sciences has been previously highlighted [10]; however, progress toward improving the current state-of-play has been slow. Davis and colleagues [10] provide an expert consensus definition of theory which reads: “A set of concepts and/or statements with specification of how phenomena relate to each other. Theory provides an organising description of a system that accounts for what is known, and explains, and predicts phenomena.” In line with this definition, theory can be associated with the evolution of scientific knowledge and the notion of an objective, explanatory lens upon the world [11, 13]. Consequently, the cumulative outcome of rigorous empirical testing and validation of theories across different settings is a series of structured frameworks in which knowledge may be coherently organised, accumulated, and advanced over time.

### Benefits and importance of theory application

Theory application is an integral component  in the design, implementation, and evaluation of behaviour change interventions [14]. While effectiveness is often the focus of arguments for or against theory [15], the benefits of theory extend beyond effectiveness by delivering clearer, more nuanced explanations of the sets of activities performed by interventionists and the resulting interactions and outcomes for the individuals and/or groups participating. Theory can advance research and practice by identifying what works, for whom, how, why, and when; thereby delivering a roadmap of how to design interventions that are more likely to achieve the desired outcomes [10, 14, 16–19]. Table 1 provides a synthesis of the known functions and benefits of theory application across the life cycle of an intervention: planning, design, implementation, and evaluation.

**Table 1:** Synthesis of theory application functions and benefits

Stage	Objective	Why … theory	How … theory	Source
Planning and design	Targeting	What factors should be targeted to elicit the desired change (or maintenance) in behaviour?	Identification of antecedents of behaviour *or* behaviour change. Isolation of mechanisms of action (e.g., mediators and moderators of behaviour *or* behaviour change) that form intervention targets.	[20–23]
Planning, design, and implementation	Mapping	Which intervention components can be used to influence intervention targets in the desired direction?	Means of selecting intervention components (activities, strategies, and behaviour change techniques) that need to be embedded and mapped within the intervention design to ensure identified mechanisms of action will be influenced in the desired direction.	[16, 23, 24]
Planning, design, and implementation	Tailoring	Who to target, with what, and when?	Interventions (activities, strategies, and behaviour change techniques) can be tailored on a one to one or group (segmented) basis by ensuring theoretically identified differences are catered for in intervention design and implementation, thereby delivering more benefit to more people.	[23, 25]
Planning, design, implementation, and evaluation	Modelling	How do behavioural determinants, intervention components, and outcomes relate to each other?	Inform the development of a logic model (road map) visually depicting the relationships between the target behaviour, determinants (mechanisms of action), intervention components, and outcomes.	[16, 26]
Evaluation	Measurement	What to monitor and measure, and how?	Provide a guide for intervention evaluation ensuring theoretically derived determinants (mechanisms of action) of behaviour (or behaviour change) and associated intervention components are monitored and measured.	[16, 23]
Evaluation	Effects	What works, for whom, how, why, and when?	Determinants can be empirically investigated to gain a further understanding as to how the intervention elicits (or not) effects via mediation analysis, and to enhance intervention effectiveness and efficiency over time.	[27, 28]
Evaluation	Reporting	How can quality and rigour be evaluated within and across studies?	Theories provide a series of organising frameworks that can support accurate and complete description of interventions.	[16]
Evaluation	Testing	What factors or combination of factors best explain the target behaviour?	Interventions provide an opportunity to test theory and contribute to the development of theories delivering stronger explanatory and predictive potential over time, which in turn, supports future intervention optimisation, enhanced outcomes, and cumulative knowledge advancement.	[11, 29]

As shown in Table 1, there are many functions and benefits of rigorous theory application and explicit reporting of theory use across the planning, design, implementation, and evaluation of behaviour change interventions. Taken together, theory provides a structured framework in which knowledge of how to change behaviour across different populations and settings can be coherently organised, accumulated, and advanced over time [10, 11, 20].

### The need to standardise theory application and reporting

Evidence for an association between theory use and increased intervention effectiveness remains mixed [15, 30, 31]. Several systematic reviews find theory-based interventions generate larger effects than interventions which do not report use of theory at all [23, 32–36]. Other reviews of interventions incorporating theoretically derived behaviour change techniques report similar associations with increased effectiveness [37–39]. Some reviews report equivocal or inconsistent support for theory-based interventions [40–46], while others suggest that theory-based interventions are less effective than interventions that do not report theory application at all [47–49]. Highlighting the need to improve levels and quality of theory use, proponents of theory have provided several explanations for this mixed picture including inappropriate selection of theory and/or combining multiple theories with no sound conceptual justification; insufficient explanatory and/or predictive power of the chosen theory; limited and/or poor use of the chosen theory; inconsistent understanding of theory; methodological limitations; failure to precisely and consistently measure theoretical constructs; and lastly, a lack of or poor reporting of theory use [15, 23, 50–52]. Moreover, evidence reviews of theory and effectiveness often deem an intervention as “theory-based” if study authors simply mention a theory, with little or no consideration given to the quality of theory use and transparency in reporting [20, 23].

Fundamentally, a theory should provide formal explanation for observed phenomena and be capable of generating potentially falsifiable predictions [53]. Informal explanation, unfalsifiable statements, and ideas are not scientific theories [54]. For those seeking to change people’s behaviour, theories should explain the ‘how, when, and why” of action or inaction [11], and they should identify the sources of influence that alter the target behaviour when applied in the desired direction and within the desired setting. The inappropriate selection of theory and/or combining of multiple theories with no sound conceptual justification is one of the commonly cited reasons for the mixed evidence surrounding the theory-effectiveness hypothesis [15, 23, 50–52]. Many theories attempting to explain or predict human behaviour have been developed across a wide range of disciplines. For example, one consensus method generated 33 theories [55]. A later scoping review identified 83 theories across the social and behavioural sciences [10]. Although a seemingly large number of theories exist, many have not been subjected to wide-scale rigorous empirical evaluation [10]. While many theories may offer valuable and relevant informal explanation, in the absence of rigorous testing and replication to determine analytical and predictive powers [19], veracity remains questionable. Moreover, practitioners cannot (and should not) use and rely on theories that have not been shown to reliably predict the target behaviour within the desired population and setting.

Empirical evaluations of theories are critical to building and refining theories over time; however, in the absence of rigorous theory application and testing, theories cannot be appropriately refuted. An appropriate refutation of a theory must be based on obtaining null effects (i.e., changing behaviour without changing theory-relevant constructs) [20]. Evidence reviews of reported theory use caution the refutation of theories based on current levels of theory application and reporting. For example, Willmott and colleague’s [23] systematic review examining reported theory use in electronic health weight management interventions targeting young adults using the 19-item Theory Coding Scheme [20] found only six (out of 24) studies measured theory-relevant constructs pre- and post-intervention, and only three reported the reliability and/or validity of the psychometric scales used to measure theory-relevant constructs/predictors. Furthermore, no study reported using intervention results to build and/or refine the theory upon which the intervention was based or formulate suggestions for future refinement. Similar findings have been reported in other evidence reviews [52, 56, 57], where theory is used to inform but not test or evaluate intervention activities and outcomes. The Transtheoretical Model (TTM) [58] provides an interesting case of a theory that has been widely applied in behaviour change research, and at the same time, has been widely criticised [59, 60]. This criticism may, in part, be attributed to limited and/or poor application of the theory. Reviews have found that researchers and practitioners applying the TTM have not applied the theory in full, with many studies focusing on the stages of change and not incorporating the key theoretical constructs proposed within the model [61]. Of note, a recent meta-analytic review found that TTM-based interventions significantly improved physical activity behaviour, and that their efficacy was moderated by the TTM theoretical constructs, not by the descriptive stages of changes that the model has become known and widely criticised for. Therefore, the TTM holds utility in terms of its theoretical constructs; however, the existence of the stages of change is questionable [59].

Theories cannot be appropriately refuted where levels of application (and reporting) are limited or poor. The Theory Coding Scheme [20] is a reliable and valid method of assessing levels and extent of theory use. Much of the scientific knowledge of how to change behaviours has been built on disparate observations or descriptions rather than explanation [11]. Given varied definitions, mixed evidence, and the fact that very few studies explicitly explain and report how theory has been applied in sufficient detail [20, 23, 62], discussions regarding the potential role of theory in enhancing intervention effectiveness remain premature and any dismissive claims should be disputed.

Dalgetty and colleagues [15] highlight the need for more standardised frameworks, methods, and processes through which to define, use, and report theory. Although progress is being made with the development of ontologies [63, 64], taxonomies [65–67], methods for mapping interventions [9, 24, 68], and coding schemes [36], evidence reviews still highlight a distinct lack of rigorous theory application and poor reporting of theory use [23, 52]. Moreover, these methods and techniques are dense and technical making it difficult for practitioners to understand how they can (and should) be applied in practice. Similarly, researchers with no or limited practical experience may overlook the importance of rigorous theory application and explicit reporting of theory use, which further limits our capacity to advance the cumulative evidence base. Therefore, consolidating existing methods and resources into a standardised framework could improve the current state-of-play by ensuring we are all “speaking the same language.” Accordingly, we call for a framework to support the standardisation of theory application and reporting in behaviour change research. A standardised framework would move the research community toward applying theory in a concrete and consistent manner and ensure reporting of theory use is transparent permitting critical appraisal within and across studies to more rapidly advance understanding. Acceptance and implementation of a standardised operating framework that researchers serially apply will further support theory building and refining over time.

### Recommendations and a call for action

Theory can be highly abstract and lack relevance to practice [63], and presently there is a lack of complete frameworks, methods, and/or processes informing theory application and reporting across the full life cycle of an intervention. In the absence of concrete and consistent application, theory cannot be reliably expected to deliver the roadmaps that practitioners need to enact the desired changes in behaviour in real-world settings. To accelerate progress in the field in terms of uptake of rigorous theory application and explicit reporting of theory use, the focus must shift to encouraging researchers and practitioners to take collective action. The lack of and/or underreporting of theory use within behaviour change research suggests that researchers and practitioners need further support in applying theories to intervention design, implementation, and evaluation. We provide four recommendations for the development of a standardised framework that will inform rigorous theory application and explicit reporting of theory use across the complete  intervention life cyle:

1. *Communicate* the benefits of theory use beyond effectiveness.
2. Acknowledge the *complexity* of behaviour.
3. *Consolidate* existing methods and resources.
4. Be *practical*.

First, the framework must leverage, and effectively communicate, the known benefits of theory use (beyond effectiveness) to researchers and practitioners. Enhanced communication would allow basic and applied behavioural scientists to recognise the strengths and weaknesses of current theories of health behaviour (and levels of application), and thus help formulate a fuller understanding of what needs to be done to improve the quality of our theories [29]. For example, ‘guiding’ is one of the more effective, albeit underutilised, styles of communication in behaviour change [64]. Guiding focuses on motivating, supporting, and/or bringing people with you [64]. Consequently, providing best practice examples, giving actionable advice, and presenting opportunities for researchers and practitioners to realise the benefits of theory use firsthand is likely to result in increased uptake and improved levels of application through enhanced communication.

Second, the framework must acknowledge the *complexity* of behaviour and the resulting complexity of the interventions designed to influence behaviour. Many theories are available for use [10, 55], all varying in their perspective and scope [65]. Behaviour change entails more than simple messaging that appeals to common sense [66]. Behaviours are embedded in complex systems involving individuals, groups, and communities operating in diverse physical and social environments. As a result, interventions intended to influence one or more behaviours are often complex and typically involve multiple intervention components and modes of delivery that may work independently or together to influence behavioural determinants and elicit desired outcomes. Most theories focus on what people think and feel rather than what interventionists do and how individuals participating in interventions respond. A significant shift in theory construction and use is needed to deliver clear roadmaps that can effectively guide practitioners. Effort must be directed toward identifying a level of standardisation that accommodates variation in intervention design and outcomes while contributing to the collective advancement of the cumulative knowledge base. Thus, the framework must provide guidance for the identification of theories which extend focus beyond the individual, and support application across different populations and settings.

Third, the framework should consolidate existing methods and resources to provide an evidence-based guide to support the rigorous application and reporting of theory use. Emerging ontologies [67, 68], taxonomies [69–71], methods for mapping interventions [9, 24, 72], and coding schemes [36] address deficiencies in theory use (and associated behaviour change techniques) during the development of behaviour interventions; however, these methods and resources are diverse and fragmented. If existing methods and resources were situated within a broader standardised framework that explained the ‘how,’ ‘when’ and ‘why’ of available methods and resources, application of theory (and associated behaviour change techniques) would occur in a more concrete and consistent manner, thereby advancing the cumulative knowledge base through the creation of a common semantic structure. The standardised framework called for is a way in which guidelines and processes can be brought together to address the fragmentation evident among existing methods and resources to ensure research can be translated into effective practice.

Lastly, the framework should be practical. Specifically, the framework must be considerate of real-world constraints, including various contextual factors (e.g., level of expertise, knowledge, skills, resources etc.) that may impact the ability of researchers and practitioners to implement any developed framework. The lack of relevance, accessibility, and applicability of theory to the rest of society has been coined the ‘practicality crisis’ [69]. Berkman and Wilson [69] highlight the need for increased pragmatism within theory construction and application. Davidoff [18] exemplify how theory may be de-mystified for researchers and practitioners who are reluctant to use theory. Accordingly, the standardised framework should support researchers to deliver processes that can be applied in practice to achieve the intended outcomes. Translation requires collaboration between theorists and interventionists. As Rothman [28] persuasively argued almost two decades ago, innovations in theory and practice require interdependence in the research activities undertaken by basic and applied behavioural scientists. The emergence of implementation science highlights the value and importance of such collaboration [17]. The delivery of frameworks, methods, and processes that support theoretical application and translation in practice are critical to providing a semantic structure; that is, a common language by which to guide the systematic development and evaluation of behaviour change interventions [17, 18, 73]. Interventions provide an invaluable opportunity to test and refine theory that should be capitalised upon to advance the cumulative knowledge base over time.

The four recommendations described above are intended to guide collective action within the health behaviour change community. Improved application and reporting of theory use will require widespread acceptance and adoption of a standardised framework. Accordingly, this paper represents a call for action and does not seek to propose an immediate and definitive solution. In line with the four recommendations outlined, the authors have made an initial attempt to articulate a standardised framework with clear processes delineated for researchers to follow in a subsequent paper. The paper titled “Developing a theory application process: An integrative review and critical analysis of theory use in health behaviour change research” provides structured evidence-based guidance that researchers can follow to rigorously apply theory to intervention design, implementation, and evaluation [74]. The authors expect that this early version of a standardised framework, like any theory, will need many applications across different settings to deliver a standardised framework that can be consistently and concretely applied across studies as called for in this paper. Standardising theory application and reporting is a significant undertaking that will require the support of the health behaviour change community including researchers, practitioners, professionals, funding bodies, and policy makers. Activities such as information sharing symposiums and/or training workshops can be used to bring the community together and present the call for action described in this paper. As researchers, it is important to consider such complementary activities, outside of peer-reviewed publications, that will support the adoption of transparent conduct and reporting practices for theory application. An information sharing symposium, for example, would provide an opportunity to motivate, support, and bring the health behaviour community together on the issue of rigorous theory application and reporting [64]. Importantly, an information sharing symposium would allow people from different backgrounds to come together to exchange knowledge and experiences, and in doing so, would likely increase acceptance and adoption of a standardised framework. Equally, training workshops offering best practice examples, actionable advice, and a contemporary perspective on the benefits of theory use would likely increase uptake of a standardised framework, thereby improving levels of application through enhanced communication and skills building. In an era where proving results from investments made continues to rise in importance, heeding this call for action will ensure theory is applied in a manner that supports intervention optimisation and the effective translation of research into health behaviour change practice thus ensuring desired outcomes are achieved.

### Closing remarks

We call for the development of a standardised framework to consolidate existing methods and resources in a complete process to improve levels and quality of theory use, provide a semantic structure, and importantly, facilitate the translation of theory-driven research in practice. A standardised framework will deliver more concrete and consistent application and reporting of theory directly addressing the practicality crisis. Importantly, developing a clearer picture of what works, for whom, how, why, and when will deliver the understanding needed to reduce preventable risks to human health, and improve individual and population health outcomes over time.

## Data Availability

Not applicable.
